# The effect of depression on the peak alpha frequency as a biomarker of pain sensitivity

**DOI:** 10.1016/j.ynpai.2025.100193

**Published:** 2025-08-06

**Authors:** Mingge Shi, Luiza Bonfim Pacheco, Natalia Egorova-Brumley

**Affiliations:** a Melbourne School of Psychological Sciences, University of Melbourne, Melbourne, Australia; b School of Psychological Sciences, Monash University, Melbourne, Australia

**Keywords:** Pain, Depression, EEG, Peak alpha frequency

## Abstract

**Objective:**

To assess how Peak Alpha Frequency (PAF) as a neurophysiological biomarker of pain sensitivity is influenced by conditions often comorbid with chronic pain, e.g., depression, and how methodological differences in deriving PAF, e.g., from Eyes-open (EO) vs. Eyes-closed (EC) EEG recordings affect this association.

**Methods:**

We analyzed data from 47 participants (70 % female) aged 18–51 years (*M* = 25.0, *SD* = 6.50). Among them, all participants underwent EO EEG recording but only a subset of 25 participants underwent both EO and EC recording. Depression (Patient Health Quotient – 9 M = 4.49, SD = 3.96) and sensitivity to heat pain were measured.

**Results:**

In EO, Spearman correlations showed no significant PAF-pain relationship (p = 0.530) but a positive correlation with depression (ρ = 0.348, p = 0.019). In EC, no significant correlations emerged, though a trend (p = 0.052) suggested depression might moderate PAF-pain links. Notably, the EO-EC PAF difference negatively correlated with depression (ρ = −0.54, p < 0.01).

**Conclusions:**

PAF may be sensitive to depression, albeit in the opposite direction to pain, and therefore mask the association between PAF and pain in individuals with depression. Differences in EO vs. EC PAF, as well as the EO-EC difference warrant further study.

**Significance:**

Depression affects PAF especially in the eyes-open recordings.

## Highlights

• Peak Alpha Frequency (PAF) from the eyes-open EEG recording positively correlates with depression.
• Depression may modulate the relationship between pain and PAF.
• Alpha reactivity is promising as a marker for depression, addressing variability in PAF from eyes-open and eyes-closed EEG.

## Introduction

Among the various neural rhythms, alpha oscillations (8–12 Hz) reflect the activity of thalamocortical circuits and play a critical role in sensory gating and attentional processes (Vijayan & Kopell, 2012). Peak alpha frequency (PAF), defined as the dominant frequency within the alpha range, is an individual-specific marker of alpha oscillatory speed and has emerged as a promising biomarker for sensation, perception, and cognitive functioning (Başar, 2012).

Recently, PAF has garnered attention as a potential biomarker for pain sensitivity, a known risk factor for the development of chronic pain conditions such as fibromyalgia, migraines, and arthritis (Chowdhury et al., 2023, Chowdhury et al., 2025, Furman et al., 2018). Individuals with heightened pain sensitivity often exhibit slower PAF compared to healthy controls, a pattern thought to reflect impaired sensory gating and increased vulnerability to pain (Furman et al., 2018, Raghuraman et al., 2019, Valentini et al., 2022). Slower PAF may signal reduced attentional flexibility, limiting the ability to suppress painful stimuli.

In parallel, PAF has also been explored as a biomarker of depression marked by persistent low mood, cognitive impairments, and attentional deficits. Unlike the slowing observed in chronic pain, faster PAF has been associated with depression, potentially reflecting heightened cortical excitability and baseline arousal states (Tement et al., 2016, Zhou et al., 2023). Depression commonly co-occurs with chronic pain (Zheng et al., 2022); up to 50 % of individuals with chronic pain also meet the criteria for major depressive disorder (IsHak et al., 2018). Yet, how PAF that exhibits opposite tendencies for pain and depression would predict pain in the presence of depression remains unclear.

Another notable gap in the current research is the limited exploration of the sensitivity of eyes-open (EO) PAF recording as a biomarker for pain sensitivity or depression. Prior studies, including those referenced above, typically used eyes-closed (EC) conditions for PAF measurement due to their higher signal-to-noise ratio and fewer artifacts (Petro et al., 2022). To extend these findings, we used EO recordings to test whether this relationship is context-dependent—specific to factors that are unique in the EC state—or reflective of a more general neurophysiological process. EC conditions may be characterized by increased alpha synchronization and greater involvement of the default mode network, potentially making them more sensitive to sensory gating deficits associated with pain (Liu et al., 2022). In contrast, EO conditions engage attentional and cognitive control networks, which may be more relevant to depressive symptomatology (Barry et al., 2007). The potential difference in sensitivity underscores the need for systematic evaluation and comparison of PAF under both EC and EO conditions.

We aimed to investigate the relationship between PAF, pain sensitivity, and depression in both EC and EO conditions in one study. By directly comparing these conditions, we sought to clarify the relationship between PAF as a biomarker of pain and depression. In line with previous research findings, we first hypothesized that PAF would be negatively correlated with pain sensitivity, with stronger effects observed in the EC condition compared to EO. For PAF’s association with depression, we hypothesized that PAF would be positively correlated with depression, with stronger effects observed in the EO condition compared to EC. Lastly, we predicted that due to differences in the direction of effect for pain vs. depression, depression would reduce the strength of the association between pain sensitivity and PAF.

In addition, we explored the relationship between the difference between EO and EC PAF in relation to both pain sensitivity and depression. By addressing these questions, this study aimed to provide new insights into the clinical applicability of PAF in chronic pain conditions comorbid with depression.

## Method

### Participants

Participants were recruited through online advertisements targeting the student population at the University of Melbourne.

Participants were eligible for inclusion if they were aged 18 to 65 years and reported diagnosed depression, self-reported depression, or no depression. Depression severity was determined based on self-report using the Patient Health Questionnaire-9 (PHQ-9). Participants with any level of depression were included, so depression was treated as a continuous variable rather than a binarized category.

Participants were excluded if they were diagnosed with pre-existing pain conditions (e.g., chronic pain syndromes, arthritis) to minimize confounding effects on pain sensitivity measures, or had suicidal ideation (measured with PHQ-9 item 9).

A total of 61 participants were initially recruited. Two were excluded due to existing pain conditions. No participants were excluded for suicidal concerns. An additional twelve participants were excluded due to missing or unsuitable EEG data, resulting from technical errors. The final sample for analysis comprised N = 47 pain-free adult participants (70 % female) aged 18 to 51 years (*M* = 25.0, *SD* = 6.50). Of these, N = 25 participants (72 % female, age range: 18–23 years, *M* = 24.08, *SD* = 4.42) underwent both EC and EO EEG recording; while N = 22 participants (64 % female, age range: 18–49 years, *M* = 25.71, *SD* = 7.66) underwent EO EEG recording only. This study was approved by The University of Melbourne Human Research Ethics Committee and carried out at the Melbourne School of Psychological Sciences Pain and Cognition Neuroimaging Lab. Informed written consent was obtained prior to participation.

### Assessing heat pain sensitivity

Thermal stimuli were delivered to the volar surface of the participant’s left forearm using a thermal contact heat stimulator (Medoc Pathway; Medoc Advanced Medical Systems Ltd., Ramat Yishai, Israel). Participants were provided with a paper copy of the Gracely Pain Intensity Scale (Gracely et al., 1978) for reporting pain intensity. The thermode surface (30 x 30 mm) in contact with the participant’s skin was maintained at a baseline temperature of 32 °C. Participants were informed that the thermode temperature would increase from and return to baseline, with this process repeated multiple times at incrementally higher temperatures. They were instructed to rate specifically the pain, rather than the heat sensation, after each stimulation using the 0 to 20 Gracely Scale. Participants were also informed that they could remove the thermode at any time.

Upon receiving a trigger, the thermode heated to a target temperature at a rate of 10 °C per second, maintained the target temperature for two seconds, and then returned to baseline at the same rate. The stimulus sequence began when the participant indicated readiness. Calibration stimuli ranged from 35 °C to 49 °C, starting with the lowest temperature and increasing incrementally by 1 °C. Each pain rating was recorded alongside its corresponding temperature. This procedure continued until the maximum temperature was reached or until the participant provided a pain rating of 12 or greater.

Pain sensitivity in this study was operationally defined as the highest temperature that elicited a pain rating of 10/20 (“mild pain”) on the Gracely Scale. This approach is justified because pain sensitivity represents the threshold at which a stimulus transitions from non-painful to painful sensation. A rating of 10 corresponds to this critical transition point, as ratings below this value represent sensations that participants do not classify as painful and are therefore inappropriate markers for pain sensitivity assessment. While traditional button-press methods have been used to capture pain detection thresholds, graded pain ratings provide good sensitivity to individual differences and accurately reflect subjective pain experience. Previous research has demonstrated strong correlations between temperatures eliciting mild pain ratings and traditional pain detection thresholds (Dias et al., 2023), supporting the validity of this approach for quantifying pain sensitivity. A similar approach was also used in Furman et al. (2020) where the temperature at pain rating of 5/10 was used in their phasic heat pain model. If no pain rating of 10 was elicited, the highest rating below 10 was recorded, e.g., if a participant rated pain as 9 at 40 °C and 11 at 41 °C, their pain sensitivity score would be recorded as 40 °C.

### Depression

The Patient Health Questionnaire-9 (PHQ-9, Spitzer et al., 1999), a brief 9-item self-report depression screen, was used to assess the severity of depressive symptoms. The PHQ-9 was derived from the clinician-administered Primary Care Evaluation of Mental Disorders, and as such, was developed specifically for use with medical patients in clinical settings. As a severity measure, the PHQ-9 score ranges from 0 to 27, with cut-off points of 5, 10, 15, and 20, indicating the thresholds for mild, moderate, moderately severe, and severe depression, respectively (Kroenke et al., 2001).

### EEG acquisition and pre-processing

EEG data was recorded using a Biosemi Active Two system (Biosemi, the Netherlands) with 64 active scalp electrodes. We grounded the recordings using common mode sense and driven right leg electrodes. We supplemented this montage with four additional electrodes: two located 1 cm from the outer canthi of each eye, and two above and below the right eye. The sampling rate was set at 512 Hz (DC-coupled with an anti-aliasing filter, −3 dB at 102 Hz). We aimed to maintain electrode offsets within ± 40 μV, though this was not always achievable.

EEG pre-processing was conducted using Brain Vision Analyzer Version 2.1.2 (Brain Products GmbH, 2017). Filters were applied using Infinite Impulse Response (IIR) zero-phase Butterworth filters (0.5Hz high-pass with time constant 0.318 and order 2; 30 Hz low-pass with order 2; 50 Hz notch). We manually identified noisy channels with signs of flat-lining or excessive artifacts, which were excluded from subsequent processing steps (re-referencing, manual artifact removal, and ocular artifact removal), and later interpolated. No participant had more than 2 noisy channels. Our complete data processing records are available on demand from the corresponding author.

Data were initially referenced to channel Cz and segmented into 1-minute epochs time-locked to the onset of “eyes-open” and “eyes-closed” cues. We then performed Independent Components Analysis (ICA) using an infomax (gradient) restricted algorithm, classic Principal Component Analysis (PCA) sphering, a convergence bound of 1E-07, and a maximum of 512 iterations. Inverse ICA was then conducted to revert the components back to individual channels, excluding components corresponding to eye blinks and horizontal eye movements.

Post-ICA, we interpolated the excluded channels using fourth-order spherical splines and re-referenced the data to the average of all scalp channels. We then segmented data into 2-second epochs with 1-second overlap. A semi-automated artifact rejection function was then used based on voltage changes which exceeded 200uV within a 200 ms interval, or less than 0.5uV within a 100 ms interval.

Finally, we converted EEG segments to frequency domain using fast Fourier transforms (100 % Hamming Window, with all segments zero-padded to achieve a 0.5 Hz resolution). We computed average amplitudes in each 0.5 Hz frequency bin across segments for both EO and EC conditions.

### Quantification of PAF

Following pre-processing, we extracted the PAF from the EEG power spectra using the Fitting Oscillations & One Over F (FOOOF) algorithm (Donoghue et al., 2020). This algorithm parameterizes neural power spectra by separating aperiodic and periodic components.

We implemented the FOOOF algorithm using the FOOOF package (version 1.1.0) in Python (version 3.11). A FOOOFGroup object was initialized with peak width limits set to [1, 13] Hz, facilitating the detection of alpha-band oscillations. We then fitted the FOOOF model to each participant’s power spectrum data across all EEG channels, considering the frequency range of 0.5 to 30 Hz. Consistent with standard definitions in the literature, we defined the alpha band as 8–13 Hz.

For each channel, we extracted the peak frequency within the alpha band using the get_band_peak_fg function from the FOOOF package. To ensure the reliability of the extracted PAF values, we calculated the goodness of fit (R-squared) for each FOOOF model. Furthermore, we computed the mean R-squared value across all channels for each participant, providing an overall measure of model fit quality.

For PAF analysis, we focused on electrodes C3, Cz, and C4, which most strongly reflected the sensorimotor component topography as observed in Furman et al. (2018). PAF from these 3 electrodes only was extracted as per our a priori hypotheses. The sensorimotor PAF value was calculated by averaging the PAF across this region of interest for each participant in both EO and EC conditions.

The FOOOF algorithm failed to identify a PAF for 2 participants in the EO condition. This could be attributed to either a genuinely absent alpha peak or a very weak alpha peak that did not stand out from the background spectrum. Consequently, these participants were excluded in a list-wise fashion from further analysis.

### Procedure

Upon registering for the study, each participant was sent an email with preparatory information for their sessions including the Plain Language Statement (PLS), and links to Qualtrics versions of the PLS, consent form, and screening questionnaires. Once they had provided informed consent and had been screened for eligibility, they were given a verbal overview of the study procedure for the session and given the opportunity to ask questions before proceeding. Participants were tested on their pain sensitivity first. After that, we invited participants to undergo 8 min of resting state EEG. The participant was informed that at task onset, they would hear a tone, at which point they were to look at a white circle presented on the monitor. After one minute, another tone would sound, at which point the participant was instructed to press any keyboard key and close their eyes for another minute. Participants were to open their eyes when they heard the tone again and thus alternate between EO and EC conditions until the task ended. The interval between the completion of pain sensitivity testing and the initiation of EEG recording procedures was approximately 25 min. This duration encompassed the setup of recording equipment and providing task instructions. This interval was deemed sufficient to minimize potential carryover effects from prior pain testing on the recording outcomes. In total, 4 min of resting state EO and 4 min of resting state EC were collected.

### Statistical analysis

To investigate the specificity of PAF as a biomarker for pain sensitivity, our primary analytical approach involved *a priori* defined correlation analyses between sensorimotor PAF, pain sensitivity, and depression, with distinct PAF deriving methods (EO and EC). When linearity and normality assumptions were violated, we conducted non-parametric Spearman’s correlation analyses. Effect sizes were evaluated based on Spearman’s rho (ρ) and Pearson’s r, with magnitudes of 0.2, 0.4, and 0.6 interpreted as weak, moderate, and strong correlations, respectively (Rosenthal, 1991). Additionally, we utilized a linear regression model to investigate the potential effect of depression on the relationship between PAF and pain sensitivity.

Further exploratory analysis was conducted to investigate correlations between pain sensitivity, depression, and alpha reactivity quantified using the following formula adapted from Wan et al. (2018):

$$\mathrm{alpha}\;reactivity=\frac{\mathrm{PAF}\;EC-PAF\;EO}{\mathrm{PAF}\;EC}$$

While Wan et al. (2018) applied this normalization approach to calculate changes in total alpha power during EC and EO transitions, we utilized this method to quantify changes in PAF between these states. As individuals exhibit distinct patterns of brain activity changes during state transitions (e.g., Douw et al., 2016), we explored whether this reactivity could also serve as a marker for pain and depression.

## Results

### Pain sensitivity and depression

The goodness of fit test indicated that FOOOF reliably captured periodic components from aperiodic background activity, with each participant’s R^2^ value exceeding 0.90.

Descriptive statistics for pain sensitivity and depression are reported in Table 1. We assessed the normality of variables using the Shapiro-Wilk test. The results indicated non-normal distributions for both pain sensitivity (*p* < 0.001) and depression (*p* < 0.001). Given these findings, Spearman’s correlation was employed in subsequent analyses involving these two variables. Spearman correlation analysis between these two variables revealed no significant correlation, *ρ* = 0.179, *p* = 0.228.

**Table 1:** Mean, standard deviations, and range for pain and depression ratings.

Variables	*M*	*SD*	Range
Pain Sensitivity	45.851 °C	3.284 °C	39–50 °C
Depression (PHQ-9)	4.489	3.961	0–19

### Spectral analysis

**Fig. 1:**
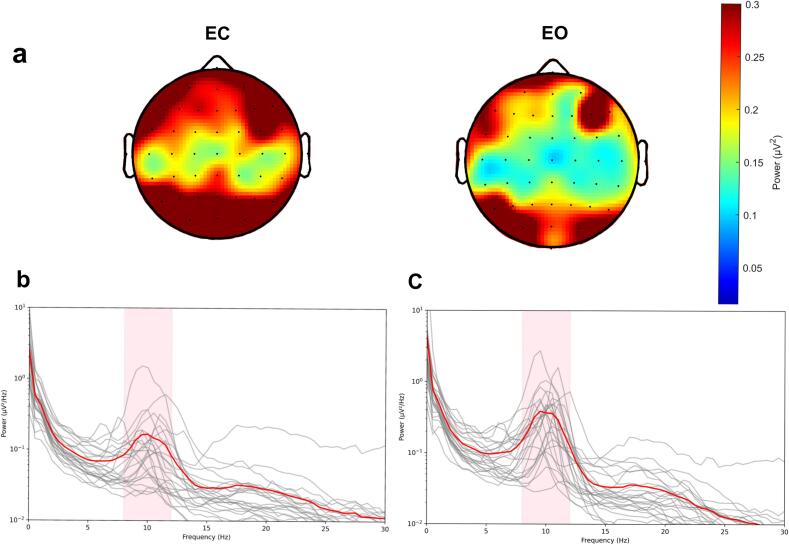
a. The topography of the Central Alpha Component selected for Peak Frequency Analysis, averaged across all participants. b. Sensorimotor ROI Spectra in EO condition. c. Sensorimotor ROI Spectra in EC condition. The plots are based on 22 recordings in the EC condition and 47 recordings in the EO condition. The gray lines in the spectra plots reflect individual participants and the red lines show the average spectra across all participants. The red shaded zone reflects the frequency range (8–13 Hz) used to calculate the PAF according to the FOOOF algorithm. (For interpretation of the references to colour in this figure legend, the reader is referred to the web version of this article.)

The topography of the central alpha component used in our analysis and the sensorimotor region-of-intertest (ROI) spectra, averaged from all participants for the EO and EC conditions, are presented in Fig. 1. The mean PAF across all participants was 10.819 Hz (*SD* = 0.840) in the EO condition and 10.803 Hz (*SD =* 0.780) in the EC condition. For participants who underwent both EO and EC recording, the PAF was highly correlated under the two conditions: Pearson *r* = 0.836, *p* < 0.001.

### Pain sensitivity, depression and PAF

**Fig. 2:**
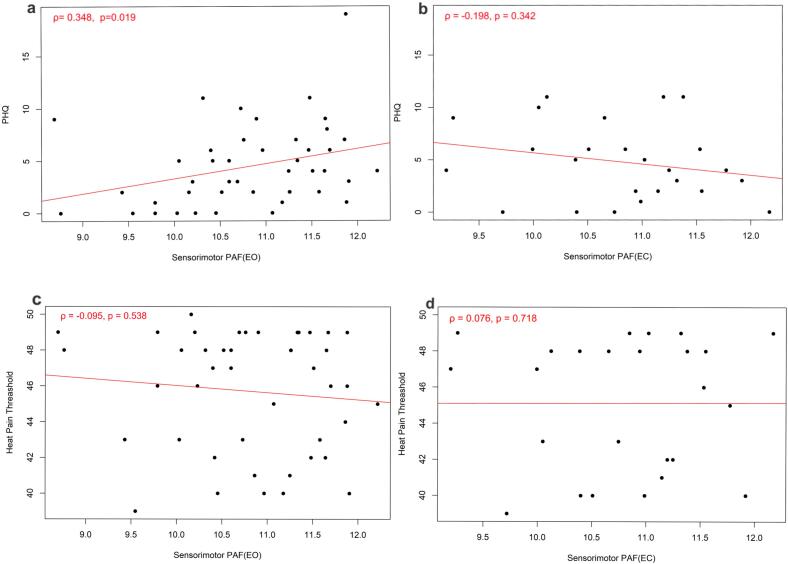
The association between PAF, Pain Sensitivity, and Depression in the EO and EC conditions. Higher PHQ-9 value reflects higher depression. Higher pain threshold reflects lower pain sensitivity.

A Spearman correlation revealed a significant positive association between depression and PAF in the EO condition, ρ = 0.348, *p* = 0.019, indicating a small to medium effect size (Fig. 2a). However, this correlation was not significant under the EC condition (Fig. 2b), ρ = −0.198, *p* = 0.342.

A Spearman correlation between the heat pain threshold and PAF showed no significant associations in either the EO condition, ρ = −0.095, *p* = 0.538 (Fig. 2c), or the EC condition, ρ = 0.076, *p* = 0.718 (Fig. 2d).

**Fig. 3:**
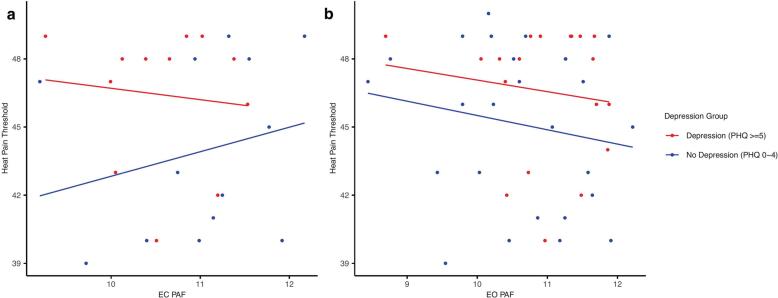
a. The relationship between EC PAF and heat pain threshold by depression status. b. The relationship between EO PAF and heat pain threshold by depression status.

Further, we tested whether the relationship between EC PAF and heat pain threshold is affected by the depression severity. A robust linear regression was conducted given the violation of normality in the heat pain threshold. Heteroscedasticity was tested by plotting the residuals by the fitted value. Upon visual inspection, no signs of heteroscedasticity were detected. The overall model was not significant, *F*(3, 21) = 2.21, *p* = 0.115, explaining approximately 17 % of the variance in heat pain threshold (pseudo *R^2^* = 0.174). Table 2 presents the unstandardized coefficients, standard errors, and t-statistics for each predictor. None of the predictors reached statistical significance at the 0.05 level, though the interaction between depression and EC PAF showed a marginal effect (b = −0.547, *t*(21) = 2.08, *p* = 0.052). The relationship between EC PAF and heat pain threshold under varying degrees of depression is illustrated in Fig. 3a. However, no such relationship was found for the EO PAF (Fig. 3b).

**Table 2:** Robust linear regression results predicting heat pain threshold.

Predictor	*b*	*SE*	*t*	*p*
Intercept	18.05	16.70	1.08	0.292
PHQ-9	5.03	2.87	1.92	0.069
EC PAF	2.35	1.53	1.54	0.138
Interaction	−0.547	0.27	2.08	0.052

**Fig. 4:**
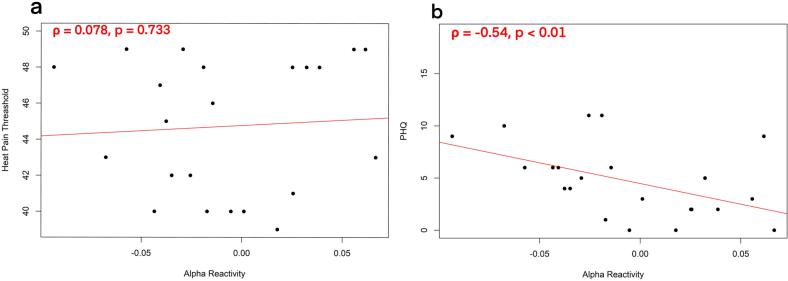
The association between alpha reactivity and a) heat pain threshold (higher threshold indicating lower sensitivity), and b) depression (higher PHQ-9 score indicating more depression).

We further explored the relationship between alpha reactivity, pain sensitivity and depression. Spearman correlation suggested that alpha reactivity and heat pain threshold were not significantly correlated, *ρ* = 0.078, *p* = 0.733 (Fig. 4a). However, alpha reactivity showed a strong association with individuals’ depression scores, *ρ* = −0.54, *p <* 0.01 (Fig. 4b).

## Discussion

We explored the relationship between PAF and pain sensitivity, a known risk factor for chronic pain, while also considering depression, as individuals with chronic pain often experience comorbid depression (Zheng et al., 2022, IsHak et al., 2018). We observed that PAF was positively correlated with depression, and importantly, we showed that the association between PAF and pain sensitivity may be affected by depression severity. Finally, we sought to understand how PAF is related to pain and depression under different resting conditions – EC and EO. We observed that EO was sensitive to depression, and that the difference between EO and EC appeared as a promising marker of depression.

Unlike prior studies reporting direct links between PAF and pain sensitivity (Furman et al., 2018, Furman et al., 2019, Furman et al., 2020; Valentini et al., 2022), our findings revealed no significant association. This discrepancy may stem from two key factors. First, depression likely moderates the PAF-pain relationship. Our inclusion of individuals with variable levels of depression revealed a trend: slower PAF correlated with higher pain sensitivity in participants with no depression, aligning with earlier work, but this relationship weakened or even reversed as depression severity increased. Though statistically non-significant this pattern suggests that depression might mask the direct PAF-pain relationship. Previous research may have overlooked this trend by examining depression and PAF as independent predictors for pain sensitivity while neglecting their interaction (e.g., Corlier et al., 2023, McLain et al., 2024). Supporting this, previous fMRI studies indicated that depression modulates pain-related activation in regions such as the ventrolateral prefrontal cortex and anterior insula (Adler-Neal et al., 2019), underscoring the need to account for depression as a confounder in future PAF-pain research.

Second, methodological differences in pain induction may explain the different findings. Furman et al., 2018, Furman et al., 2019, Furman et al., 2020 employed chronic pain-like models where capsaicin or nerve growth factor injection were used to induce long-lasting pain experience. Under this condition, prolonged nociceptor activation triggers peripheral sensitization (via sustained substance-P release; Zieglgänsberger, 2019) and central sensitization (via spinal/brain hyperexcitability; Rocha et al., 2007). Although Furman et al. (2020) also applied phasic heat pain stimuli, their 40-second stimulation duration more effectively mimicked chronic pain compared to our brief, predictable heat stimuli with incremental temperatures, which may not have adequately probed pain sensitivity. Short-duration stimuli avoid sensitization (Staud, 2010), while predictability reduces salience and novelty — factors known to diminish pain intensity by up to 25 % compared to unexpected pain (Pomares et al., 2011). Furthermore, repeated exposure may induce neural habituation, decreasing nociceptor firing rates and further constraining variability in pain ratings (Nickel et al., 2014). The resultant sensory attenuation likely limited our ability to detect individual differences in pain sensitivity, obscuring potential correlations with PAF. In a similar study where brief painful laser stimuli were used, non-significant results between PAF and pain sensitivity were similarly reported (May et al., 2025). Short-lasting pain induction is widely used in behavioral pain studies, which prioritize precise control over stimulus intensity and timing, reducing confounds from sensitization and enabling direct comparisons across participants (e.g., Castellote and Valls-Solé, 2019). As such, our brief, phasic heat stimuli lack ecological validity, as they do not reflect the sustained or dynamic nature of clinical pain or engage mechanisms like central sensitization, which are critical in chronic pain conditions. Future studies should employ pain induction paradigms that better mimic real-life pain experiences to clarify PAF-pain mechanisms in the context of depression.

In contrast to prior work (Tement et al., 2016, Zhou et al., 2023), we observed a significant positive association between depression severity and PAF in the EO condition, but not in the EC condition. The discrepancy is potentially due to the lack of power in our EC samples, given EC and EO PAF are highly correlated in our results. Note that the lack of a significant correlation between EC PAF and depression severity in a similarly modest sample of MDD patients (N = 22) was previously reported (Jiang et al., 2016). Nevertheless, the positive association we discovered in the EO condition aligns with evidence linking accelerated PAF to cortical hyperarousal, reflecting heightened neural excitability in depression (Nicholson et al., 2016, Varkevisser et al., 2005). Such hyperactivity may correspond to elevated brain metabolism, as PAF correlates with cerebral blood flow and hemoglobin oxygenation (Jann et al., 2010, Van der Worp et al., 1991)— a notion supported by positron emission tomography studies demonstrating increased thalamic metabolism in depressed individuals (Holthoff et al., 2004). Therapeutically, transcranial alternating current stimulation (tACS) protocols slowing alpha oscillations alleviate depressive symptoms (Alexander et al., 2019, Riddle et al., 2022), further implicating hyperarousal as a targetable mechanism.

A potential driver of this hyperactivity may involve the default mode network (DMN) dysregulation. Chronic DMN overactivity, characteristic of depression, is tied to maladaptive self-referential rumination (Sheline et al., 2009) and correlates with accelerated alpha oscillations and global metabolic increases (Clancy et al., 2022, Knyazev et al., 2011). Similar PAF elevations occur in conditions with DMN abnormalities, such as attention-deficit/hyperactivity disorder (ADHD) and bipolar disorder (Atagün, 2016, Slater et al., 2022), suggesting a shared mechanism of impaired network inhibition. However, resting-state EEG cannot directly assess functional connectivity; future fMRI studies are needed to clarify links between PAF acceleration and DMN hyperactivity.

While our findings implying cortical hyperarousal appear to contrast with neuroimaging reports of frontal hypoactivity in depression (e.g., Cao et al., 2016), this discrepancy likely reflects regional heterogeneity in the neural correlates of depression (Fitzgerald et al., 2008, Palmer et al., 2015). For instance, hypoactivity in frontal regions aligns with cognitive deficits (Penfold et al., 2015), whereas our central-derived PAF measures may capture hyperarousal in sensorimotor networks (Yin et al., 2016)—a pattern consistent with heightened activation in interconnected regions like the striatum and amygdala (Lyu et al., 2024, Zhong et al., 2011). Such region-specific dynamics are supported by EEG source-localization studies showing divergent excitability patterns across brain areas (Shokouh Alaei et al., 2023). Thus, depression may involve coexisting hypoactive and hyperactive states depending on the functional networks assessed. Whole-brain analyses are critical to disentangle these spatially distinct effects.

Our results identified a positive association between PAF and depression severity exclusively in the EO condition, supporting claims that EO resting state maybe better suited for probing depression-related neural activity (Ingram et al., 2024, Liu et al., 2022). This preference may stem from distinct neurophysiological mechanisms in the EO state. Visual input during EO reduces alpha power due to heightened arousal and sensory processing demands (Del Percio et al., 2011, Fonseca et al., 2013), which shifts neural activity toward externally oriented attention systems (Sadaghiani et al., 2010, Suffczynski et al., 2001). This state enhances functional connectivity in frontoparietal and dorsal attention networks—systems critical for cognitive control and emotion regulation (Agcaoglu et al., 2019, Agcaoglu et al., 2020, Zou et al., 2009). Since depression is characterized by attentional biases and emotional dysregulation, engagement of these networks during EO resting state may amplify depression-specific abnormalities, such as impaired attentional disengagement from negative stimuli. There are also differences between the EO and EC states in terms of alertness and arousal. With previous studies suggesting that depression is associated with decreased alertness (Schock et al., 2011), possibly linked to poor sleep (Kolar & Kolar, 2021), the EO PAF correlation with depression could reflect variations in arousal and attentional states rather than a direct neurobiological link. Whether direct or indirect, EO-derived EEG features outperform EC measures in classifying depression. For instance, (Liu et al., 2022) achieved optimal machine learning accuracy for first-episode depression diagnosis using EO resting-state data, likely due to its richer representation of attention-related pathology. Our findings, combined with this literature, suggest that EO and EC states tap into divergent neural processes: EO emphasizes externally oriented cognitive control, while EC favors internally focused introspection (Agcaoglu et al., 2020). Thus, the choice between EO and EC might need to be aligned with study objectives—EO for attention/emotion regulation deficits, EC for self-referential processing.

Our findings not only reaffirm the predictive value of EO PAF for depression but also highlight alpha reactivity—the PAF difference between EC and EO states—as a promising biomarker. A key strength of alpha reactivity lies in its context-independent nature. Unlike PAF, whose predictive utility appears to vary with recording condition (EC vs. EO), alpha reactivity inherently accounts for both states, mitigating the limitation of PAF studies: inconsistent methodologies (e.g., EC vs. EO) that introduce variability in results and interpretation. By quantifying the neural shift between internally focused (EC) and externally oriented (EO) states, this difference-based metric may more directly capture dynamic network reconfiguration processes relevant to depression. Specifically, alpha reactivity could reflect the brain’s capacity to adapt to changing cognitive demands—a mechanism often impaired in depression. This positions alpha reactivity as a promising measure of neural flexibility and a biomarker for depression.

This relationship between alpha reactivity and depression may be explained by the functional role of the salience network (SN). The SN has been conceptualized as a dynamic neural switch that modulates functional connectivity across various brain networks during transitions between EC and EO states (Han et al., 2023). In this context, alpha reactivity—quantified as the degree of alpha activity change between EC and EO conditions— may reflect the SN’s efficiency in orchestrating network reconfigurations, indicating the brain’s flexibility to dynamically adjust functional connectivity in response to different cognitive states.

In the context of depression, SN is critical in regulating adaptive shifts between internally focused and externally oriented cognitive states. Greater alpha reactivity likely reflects enhanced SN functionality, enabling efficient switching between the DMN, which supports self-referential processes like rumination, and the FPN, which facilitates goal-directed attention (Menon, 2011, Uddin, 2015). In depression, chronic rumination—characterized by persistent, inflexible focus on negative internal states—has been linked to SN inefficiency and failure to disengage the DMN (Hamilton et al., 2015). Reduced alpha reactivity in individuals with depression may thus signify impaired SN-mediated inhibition of the DMN during EO states, perpetuating maladaptive attention to distressing thoughts.

The inverse correlation between alpha reactivity and depression severity may further reflect stress-induced network dysregulation. Chronic stress, a known risk factor for depression, amplifies cortisol release, which impairs SN connectivity and stabilizes maladaptive DMN dominance (Liston et al., 2009). Individuals with greater alpha reactivity could have resilience against such effects, as efficient SN functionality may buffer stress-related neural rigidity. This parallels observations that non-depressed individuals exhibit stronger SN-mediated DMN suppression during attention tasks (Hamilton et al., 2015), a mechanism potentially quantified by alpha reactivity.

While these findings suggest a promising neurophysiological pathway, the SN’s role should be tested directly through multimodal imaging. For example, simultaneous EEG-fMRI could clarify whether alpha reactivity correlates with SN-DMN-FPN activation patterns during EC/EO transitions. Additionally, individual differences in stress reactivity (e.g., cortisol profiles) may modulate alpha dynamics, warranting further study.

Despite providing critical insights into the relationship between PAF, pain sensitivity, and depression, this study has several limitations. Firstly, due to practical constraints, the sample size for the present study was not optimal. This may have increased the likelihood of Type II errors (Williams et al., 1997), contributing to the non-significant findings observed, particularly regarding the close but non-significant moderation effect of depression on the association between PAF and pain sensitivity. Replication studies with larger samples are necessary to improve statistical power and validate our findings.

While this study revealed the critical relationship between PAF, pain sensitivity, and depression using EEG, future work could extend these findings by further investigating the neural mechanisms underlying this relationship. EEG was chosen for its high temporal resolution and established utility in capturing dynamic neural oscillations like PAF, which are critical for probing real-time cortical excitability and arousal states. However, as with all methodologies, EEG has inherent trade-offs: its spatial resolution limits precise source localization. Nevertheless, the widespread distribution of PAF-pain associations across scalp channels may suggest the involvement of distributed networks or subcortical generators, such as the thalamus (Behrens et al., 2003, Furman et al., 2019, Hughes and Crunelli, 2005). Future studies integrating EEG with spatially sensitive techniques (e.g., fMRI, source localization) would further clarify whether these relationships reflect thalamocortical interactions, frontoparietal network dynamics (Sadaghiani et al., 2012), or other large-scale systems.

## CRediT authorship contribution statement

**Mingge Shi:** Writing – review & editing, Writing – original draft, Software, Methodology, Formal analysis, Data curation, Conceptualization. **Luiza Bonfim Pacheco:** Writing – review & editing, Formal analysis, Data curation. **Natalia Egorova-Brumley:** Writing – original draft, Supervision, Resources, Project administration, Funding acquisition, Conceptualization.

## Declaration of competing interest

The authors declare that they have no known competing financial interests or personal relationships that could have appeared to influence the work reported in this paper.
